# Checklist of rodents and insectivores of the Crimean Peninsula

**DOI:** 10.3897/zookeys.948.51275

**Published:** 2020-07-13

**Authors:** Nikolay N. Tovpinets, Igor L. Evstafiev, Valeriy V. Stakheev, Andrey A. Lissovsky

**Affiliations:** 1 Centre of Hygiene and Epidemiology in the Republic of Crimea and city of Sevastopol, Russia Centre of Hygiene and Epidemiology Sevastopol Russia; 2 The Southern Scientific Centre of the Russian Academy of Sciences, Rostov-on-Don, Russia The Southern Scientific Centre of the Russian Academy of Sciences Rostov-on-Don Russia; 3 Zoological Museum of Moscow State University, Moscow, Russia Zoological Museum of Moscow State University Moscow Russia

**Keywords:** Crimean Peninsula, insectivores, rodents, spatial distribution

## Abstract

A dataset comprising 6806 records is presented of 17 (of total 24) rodent and insectivore species from the Crimean Peninsula collected during a 35-year period. All records are stored in the Public Mammal Database (Mammals of Russia; http://rusmam.ru/). The density of occurrence points allows visual evaluation of species distribution, even on large-scale maps. Each record contains the species name, locality description, and geographic coordinates, coordinate accuracy, date and author of the record, data source, and the method of species identification.

## Introduction

Small mammals [in particular, Rodentia (rodents) and Eulipotyphla (insectivores)] represent one of the most substantial components of the majority of terrestrial ecosystems. Being among the most diverse and abundant mammalian orders, rodents and insectivores play a critical role in maintaining the ecosystem. They also serve as reservoirs of many infectious diseases of humans, livestock, and wildlife being thus important from the perspective of public health (Evstafiev 2017). It is not surprising that studies of rodent and insectivore diversity and distribution have a long history.

Crimean fauna is heterogeneous and consists of two sharply different groups of species, steppe and mountain (Puzanov 1949). Steppe species penetrated Crimea from the northeast of Black Sea region recently. Mountain fauna is rather autochthonous. Crimean Mountains provided refuge for forest related species during the last glaciation cycle (Markova 2011).

The history of mammalogical studies in the Crimean Peninsula has earlier been described by Dulitskiy (2001a, 2001b), whereas the general characteristics of the mammalian fauna of the Peninsula can be found in Nikolskiy (1891), Puzanov (1927), and Vshivkov (1966). However, information on rodents and insectivores provided in these publications is purely descriptive. More detailed account of the distribution, ecology, and medical and agricultural importance of these animals in the Crimean Peninsula has been reported by Tovpinets and Evstafiev (Tovpinets and Evstafiev 2010; Tovpinets 2012; Evstafiev 2015, 2016, 2017). However, these publications did not present specific data on all known localities for a given species, while maps and observation lists have geographical uncertainty and lack time references.

Here, we publish a checklist of rodent and insectivore records across the Crimean Peninsula for the first time. This checklist was based on comprehensive surveys of small mammals carried out from 1983 until 2018.

Insectivores are represented in Crimea by six species belonging to two families (Dulitskiy 2001a).

**Family Erinaceidae Fischer, 1814**

1. Northern white-breasted hedgehog Erinaceus roumanicus Barrett-Hamilton, 1900

**Family Soricidae Fischer, 1814**

2. Eurasian pygmy shrew Sorex minutus Linnaeus, 1766
3. Caucasian pygmy shrew Sorex volnuchini Ognev, 1921
4. Mediterranean water shrew Neomys anomalus Cabrera, 1907
5. Bicolored white-toothed shrew Crocidura leucodon Hermann, 1780
6. Lesser white-toothed shrew Crocidura suaveolens Pallas, 1811

Rodents are represented by 18 species belonging to 5 families.

**Family Sciuridae Fischer, 1817**

1. Red squirrel Sciurus vulgaris Linnaeus, 1758
2. Pygmy ground squirrel Spermophilus pygmaeus Pallas, 1778

**Family Sminthidae Brandt, 1855**

3. Southern birch mouse Sicista lorigera Nordmann, 1839

**Family Allactagidae Vinogradov, 1925**

4. Great jerboa Allactaga major Kerr, 1792

**Family Cricetidae Fischer, 1817**

5. Gray dwarf hamster Cricetulus migratorius Pallas, 1773
6. Common hamster Cricetus cricetus Linnaeus, 1758
7. Muskrat Ondatra zibethicus Linnaeus, 1766
8. Northern mole vole Ellobius talpinus Pallas, 1770
9. Common vole Microtus arvalis Pallas, 1778
10. East European vole Microtus rossiaemeridionalis Ognev, 1924
11. Social vole Microtus socialis Pallas, 1773

**Family Muridae Illiger, 1811**

12. Pygmy wood mouse Sylvaemus uralensis Pallas, 1811
13. Steppe wood mouse Sylvaemus witherbyi Thomas, 1902
14. Yellow-necked wood mouse Sylvaemus flavicollis Melchior, 1834
15. House mouse Mus musculus Linnaeus, 1758
16. Mound-building mouse Mus spicilegus Petenyi, 1882
17. Norway rat Rattus norvegicus Berkenhout, 1769
18. Black rat Rattus rattus Linnaeus, 1758

Six species (*Erinaceus roumanicus*, *Sciurus vulgaris*, *Spermophilus pygmaeus*, *Allactaga major*, *Ondatra zibethicus*, and *Ellobius talpinus*) reported earlier for the Crimean Peninsula (Dulitskiy 2001a) have not been detected during our surveys because our methods are inadequate for these species. Two of them (*Sciurus vulgaris* and *Ondatra zibethicus*) have been recently introduced to the Crimean Peninsula (Dulitskiy 2001a).

In general, rodent and insectivore fauna of the Crimean Peninsula is depauperated. For instance, some species that are common in neighboring regions with similar environment such as Taman Peninsula and the northeast of Black Sea coast, are absent from Crimea. These include shrews of the superspecies *Sorex araneus* Linnaeus, 1758, the greater blind mole rat *Spalax microphthalmus* Güldenstädt, 1770, and Strands’s birch mouse *Sicista strandi* Formosov, 1931 (Stakheev et al. 2017). Species, which are common in the Caucasus Mountains are also absent in mountainous Crimea (voles of the *Terricola* subgenus, and the dormice *Dryomys nitedula* Pallas, 1778 and *Glis glis* Linnaeus, 1766). However, the Caucasian pygmy shrew *Sorex volnuchini* has recently been found in Crimea (Vega et al. 2020) but we have no information on this species in the current study.

From an ecological perspective, xerophilous species comprise the largest group, it includes nine species. Some xerophiles (*Spermophilus pygmaeus*, *Allactaga major*, *Cricetulus migratorius*, *Ellobius talpinus*, *Microtus socialis*, and *Mus spicilegus*) occur only in plains and submontane habitats, whereas other species (*Sylvaemus witherbyi* and *Crocidura leucodon*) invade mountains as well.

Dendrophile rodents and insectivores are represented by four species only (*S. vulgaris*, *Neomys anomalus*, *Sylvaemus flavicollis*, and *Sylvaemus uralensis*). Of them, the only true arboreal species *S. vulgaris* is not an aboriginal Crimean species but has been introduced to the peninsula.

A large group of species is associated with human settlements. Eleven species (*Crocidura suaveolens*, *Sylvaemus witherbyi*, *Sylvaemus uralensis*, *Sylvaemus flavicollis*, *Mus musculus*, *Rattus norvegicus*, *Rattus rattus*, *Microtus obscurus*, *Microtus socialis*, *Cricetus cricetus*, *Cricetulus migratorius*) have repeatedly been recorded in residential areas (Evstafiev 2016). However, only three species, the house mouse *Mus musculus*, Norway *Rattus norvegicus* and black rats *Rattus rattus*, are truly commensal. In addition, the common hamster *Cricetus cricetus* is often found in urban environment, e.g., from the outskirts to the central regions of the city of Simferopol (Surov et al. 2016).

## Taxonomic coverage

The dataset contains 6806 records of rodent and insectivore species from the Crimean Peninsula (Table 1).

**Table 1.** Number of records of rodents and insectivores collected in the Crimean Peninsula.

№	Species	Number of records
1	Sorex cf. minutus (*S. minutus* or *S. volnuchini*)	42
2	*Neomys anomalus*	10
3	*Crocidura leucodon*	108
4	*Crocidura suaveolens*	649
5	*Sicista lorigera*	38
6	*Cricetulus migratorius*	337
7	*Cricetus cricetus*	9
8	*Microtus socialis*	787
9	*Microtus arvalis*	7
10	*Microtus rossiameridionalus*	3
11	Microtus cf. arvalis (*M. arvalis* and *M. rossiameridionalus*)	571
12	*Sylvaemus uralensis*	579
13	*Sylvaemus witherbyi*	2021
14	*Sylvaemus flavicollis*	308
15	*Mus musculus*	1082
16	*Mus spicilegus*	247
17	*Rattus norvegicus*	7
18	*Rattus rattus*	1

## Taxonomic ranks

**Kingdom**: Animalia

**Phylum**: Chordata

**Class**: Mammalia

**Order**: Eulipotyphla, Rodentia

**Family**: Erinaceidae, Soricidae, Sminthidae, Cricetidae, Muridae

**Genus**: *Sorex*, *Neomys*, *Crocidura*, *Sicista*, *Cricetulus*, *Cricetus*, *Microtus*, *Sylvaemus*, *Mus*, *Rattus*

**Species**: Sorex cf. minutus, *Neomys anomalus*, *Crocidura leucodon*, *Crocidura suaveolens*, *Sicista lorigera*, *Cricetulus migratorius*, *Cricetus cricetus*, *Microtus socialis*, *Microtus arvalis*, *Microtus rossiameridionalus*, *Sylvaemus uralensis*, *Sylvaemus witherbyi*, *Sylvaemus flavicollis*, *Mus musculus*, *Mus spicilegus*, *Rattus norvegicus*, *Rattus rattus*

## Spatial coverage

The data set covers the entire Crimean Peninsula. Coordinate box: 44°23'N to 46°13'N Latitude; 32°28'E to 36°38'E Longitude.

## Temporal coverage

The data were collected from 1983 to 2018.

## Method

The major part of the data set was obtained during epizootiological survey of the Crimean Peninsula. Mammals were captured using small spring snap-traps (120 × 55 mm) deposited for one night in a line of 50–100 traps with a distance of 5 m between them and baited with bread and sunflower oil. The voucher specimens are stored in the personal collection N. Tovpinets, Simpheropol (zootonik@gmail.com). Data on *Cricetus cricetus*, *Rattus norvegicus*, and *Rattus rattus* were obtained via direct observations and/or detection of the traces of their activities (tracks, burrows, etc.).

## Dataset description

Each record contains species name after Lissovsky et al. (2019), geographic coordinates, description of locality and habitat, coordinate accuracy (in meters), date and author of the record, data source (museum specimen, photo availability etc.), type of information used for species identification (morphology, cytogenetics, genetics, etc.), and relative abundance per 100 traps/nights.

The dataset is compiled in the public database ‘Mammals of Russia’ (http://rusmam.ru/; Lissovsky et al. 2018), where all records are validated by experts.

**Character encoding**: UTF-8;

**Language**: Russian/English;

**License**: Creative Commons Attribution 4.0 International = CC BY 4.0;

**Digital identifiers**: http://rusmam.ru/sample/records?id=2_b9486

## Supplementary materials

Supplementary material 1 Cadastre of Rodents and Insectivores of the Crimean Peninsula Data type morphological, genetic Explanation note The dataset contains 6806 records of 17 (out of 23) rodent and insectivore species from the Crimean Peninsula, collected during a 35-year period mainly during epizootological surveys of the peninsula. https://binary.pensoft.net/file/431538This dataset is made available under the Open Database License (http://opendatacommons.org/licenses/odbl/1.0/). The Open Database License (ODbL) is a license agreement intended to allow users to freely share, modify, and use this Dataset while maintaining this same freedom for others, provided that the original source and author(s) are credited.Nikolay N. Tovpinets, Igor L. Evstafiev, Valeriy V. Stakheev, Andrey A. Lissovsky
